# Silent cerebral infarct after cardiac catheterization as detected by diffusion weighted Magnetic Resonance Imaging: a randomized comparison of radial and femoral arterial approaches

**DOI:** 10.1186/1745-6215-8-15

**Published:** 2007-06-07

**Authors:** Michèle Hamon, Francesco Burzotta, Catherine Oppenheim, Rémy Morello, Fausto Viader, Martial Hamon, the SCIPION Investigators

**Affiliations:** 1 Department of Radiology, University Hospital of Caen, Normandy, France; 2 Department of Cardiology, Catholica University Hospital, the Sacred Heart, Roma, Italy; 3 Department of Neuroradiology, Sainte-Anne Hospital, Paris Descartes University, Paris, France; 4 Department of Statistics, University Hospital of Caen, Normandy, France; 5 Department of Neurology, University Hospital of Caen, Normandy, France; 6 Department of Cardiology, University Hospital of Caen, Normandy, France; 7 **S**ilent **C**erebral **I**nfarct and **P**ercutaneous Cardiovascular **I**ntervention Evaluati**ON**

## Abstract

**Background and objective:**

Cerebral microembolism detected by transcranial Doppler (TCD) occurs systematically during cardiac catheterization, but its clinical relevance, remains unknown. Studies suggest that asymptomatic embolic cerebral infarction detectable by diffusion-weighted (DW) MRI might exist after percutaneous cardiac interventions with a frequency as high as 15 to 22% of cases. We have set up, for the first time, a prospective multicenter trial to assess the rate of silent cerebral infarction after cardiac catheterization and to compare the impact of the arterial access site, comparing radial and femoral access, on this phenomenon.

**Study design:**

This prospective study will be performed in patients with severe aortic valve stenosis. To assess the occurrence of cerebral infarction, all patients will undergo cerebral DW-MRI and neurological assessment within 24 hours before, and 48 hours after cardiac catheterization and retrograde catheterization of the aortic valve. Randomization for the access site will be performed before coronary angiography. A subgroup will be monitored by transcranial power M-mode Doppler during cardiac catheterization to observe cerebral blood flow and track emboli. Neuropsychological tests will also be recorded in a subgroup of patients before and after the interventional procedures to assess the impact of silent brain injury on potential cognitive decline. The primary end-point of the study is a direct comparison of ischemic cerebral lesions as detected by serial cerebral DW-MRI between patients explored by radial access and patients explored by femoral access. Secondary end-points include comparison of neuropsychological test performance and number of microembolism signals observed in the two groups.

**Implications:**

Using serial DW-MRI, silent cerebral infarction rate will be defined and the potential influence of vascular access site will be evaluated. Silent cerebral infarction might be a major concern during cardiac catheterization and its potential relationship to cognitive decline needs to be assessed.

**Study registration:**

The SCIPION study is registered through National Institutes of Health-sponsored clinical trials registry and has been assigned the Identifier: NCT 00329979.

## Background

Acute cerebral infarction following cardiac catheterization is rare [1-3], but silent brain injury could occur at an unexpectedly high rate [3-6] as detected by diffusion-weighted (DW) magnetic resonance imaging (MRI). Indeed the high sensitivity of DW-MRI suggests that this technique could allow an improved estimate of cerebral ischemic events associated with cardiovascular catheterization procedures and could be used therefore as a surrogate endpoint for risk assessment of stroke [7]. The use of transcranial Doppler (TCD) sonography allows the recording of microemboli entering the middle cerebral artery during various endovascular interventions, including cardiac catheterization [8,9]. In the case of coronary bypass surgery, evidence indicates that microembolism might be related to cognitive impairment, based on the results of neuropsychological testing [10]. During cardiac catheterization, cerebral microembolism as detected by TCD has frequently been observed, but whether it is clinically relevant remains unknown [8,9]. However, recent studies mentioned above, have suggested that some of these microemboli could be related to silent cerebral embolisms responsible for acute ischemic brain injury, as documented by DW-MRI [3-6]. Furthermore there is increasing evidence that cumulative burden of ischemic brain injury causes neuropsychological deficits or aggravates vascular dementia [11]. Thus DW-MRI emerges as a valuable diagnostic method for the monitoring of periprocedural ischemic events in the brain and could be considered as a surrogate parameter for optimising diagnostic and therapeutic vascular interventions.

In the present trial two vascular access sites (trans-radial approach [TRA] and trans-femoral approach [TFA]) routinely used for cardiac catheterization and percutaneous cardiovascular interevention (PCI) will be compared for the risk of silent brain injury occurrence. Indeed TRA is associated with fewer local complications and it has been suggested that in particular right TRA, which avoids crossing the aortic arch could reduce the risk of mobilization of atherosclerotic plaque, present particularly within the aortic arch as documented previously by several authors [12-14]. However this risk of brain injury in relation to vascular access site remains largely debatable. Using DW-MRI as a surrogate parameter for brain injury assessment we propose a prospective evaluation of the occurrence of silent brain lesions in patients randomized either to TRA or TFA.

## Design and methods

### Study design

Prospective multicenter randomized trial (4 centers currently involved: 2 in France, 1 in Italy and 1 in Austria).

### Study objectives

1. Assessment in a prospective multicenter trial of the rate of silent brain injury after cardiac catheterization

2. Comparison of the TRA and the TFA on the occurrence of new cerebral lesions as detected by DW-MRI

3. Implication of silent brain infarction(s) on potential cognitive impairment

### End points

#### (i) Primary end-point

Number of patients observed with new cerebral lesions either symptomatic or not after cardiac catheterization as detected by DW-MRI in each group (comparison of radial and femoral access).

#### (ii) Secondary end-points

Impact of potential silent cerebral infarction on cognitive impairement.

Number of microembolism signals observed at TCD between the two groups.

### Study population

Patients with severe aortic stenosis scheduled for cardiac catheterization because of aortic valve stenosis to assess coronary artery tree and aortic valve disease before surgery will be prospectively and consecutively invited to participate in the study. After informed consent is obtained the patient will be included. Exclusion criteria will be a contraindication to MRI or inability to give written informed consent.

### Cardiac catheterization

All patients will be examined clinically by a senior physician and assessed for any history of previous cerebral embolism. Transthoracic echocardiography, 12-lead surface electrocardiogram, and coronary angiography will be performed for all patients. Cardiac catheterizations will be undertaken by expert interventional cardiologists using a standard Seldinger technique using 5 French (F) catheters either by TRA or TFA according to randomization assignment. Sheaths will be removed immediately after the procedure in all patients. Unfractionated heparin (50 iu/kg) will be given intravenously to all patients at the beginning of the procedure. Retrograde catheterization of aortic valve will be attempted in a right oblique projection using a long exchange guidewire (0.035 inch, 260 cm length to ensure exchange of the pigtail catheter) using a left amplatz 1 catheter or a right Judkins catheter [15]. During attempts to cross the aortic valve, the wire will be regularly withdrawn and cleaned and the catheter flushed every 2 minutes according to Grossman's recommendations [16]. When the pigtail catheter is placed in the left ventricle, the wire will be withdrawn and the catheter vigorously aspirated and pressure measurements performed. After left ventriculography, the catheter will be rapidly withdrawn from the left ventricle into the ascending aorta with simultaneous pressure measurements. Maximum and mean pressure gradients will be established. The duration of the whole procedure and fluoroscopic time in all patients will also be recorded.

### Magnetic resonance imaging

**Figure 1:**
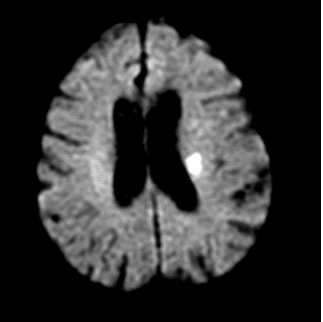
Typical positive DW-MRI (diffusion weighted-MRI) in asymptomatic patient confirming a recent cerebral infarction before catheterization.

**Figure 2:**
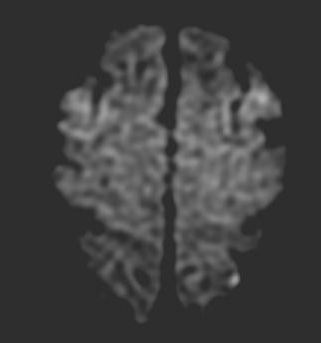
Typical positive DW-MRI (diffusion weighted-MRI) in asymptomatic patient confirming a new lesion appeared after cardiac catheterization.

MRI will be done within 24 hours before, and 48 hours after, cardiac catheterization. MRI will be performed with a 1.5 Tesla system. The imaging protocol will include a DW single-shot spin echo echoplanar sequence acquired in the Anterior Commissure-Posterior Commissure plane with 24 contiguous sections (diffusion gradient b values of 0 and 1000 s/mm^2^, repetition time TR 6000 ms, echo time TE 120 ms, slice thickness 6 mm with no gap, matrix of 128 × 128 pixels, and field of view of 240 mm); fluid attenuated inversion recovery (FLAIR; TR/TE 10000/160 ms, TI 2200 ms); and T2-weighted turbo spin echo sequences (TR/TE 3500/94 ms). For DW-MRI, the diffusion gradients will be successively and separately applied in three orthogonal directions for a total acquisition time of 24 seconds. Trace images will then be generated and apparent diffusion coefficient (ADC) maps calculated with a dedicated software tool available on all recent MRI machines. The image analysis will be performed independently by two experienced neuroradiologists who will be blinded to the clinical data and will be unaware of the technical aspects of the angiographic cardiac procedure. For analysis of DW-MRI, the neuroradiologists will be asked to determine the presence, size, number, and vascular distribution of any focal diffusion abnormalities (bright lesions) in a pattern consistent with embolic lesions. Typical positive DW-MRI showing bright lesions (cerebral infarct) in asymptomatic patients are shown in Figures 1 and 2.

### Transcranial Doppler

Transcranial Doppler studies for this work will be performed in a subgroup of patients with the TCD power M-mode Doppler (PMD) 100 (Spencer Technologies, Seattle, WA), which calculates a PMD image concurrently with a single-gate spectrogram as previously described with a threshold of 2 MHz [17]. Bilateral power M-mode TCD will be recorded during cardiac catheterization through the temporal bones of patients with an automatic detection of microemboli. The PMD image shows depth from the probe on the vertical axis, time on the horizontal axis, and power of the Doppler shift signal at specific depths as color intensity. Simultaneously, the direction of blood flow is derived from the positive or negative mean value of the Doppler shift signal at the particular depth. This mean velocity information is used in the PMD image as red, depicting flow toward the probe, and blue as flow away from the probe.

**Figure 3:**
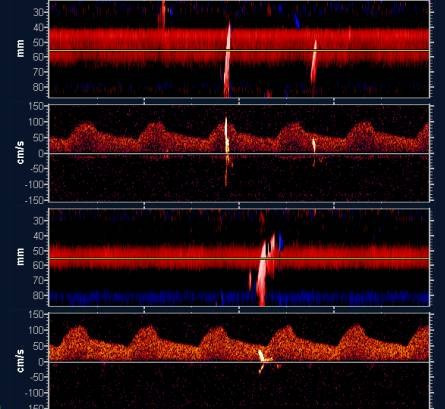
Typical microembolism signals (MES) recorded during cardiac catheterization. Isolated MES during catheter manipulation. By convention in our center upper part is for right carotid system and lower part for the left side.

**Figure 4:**
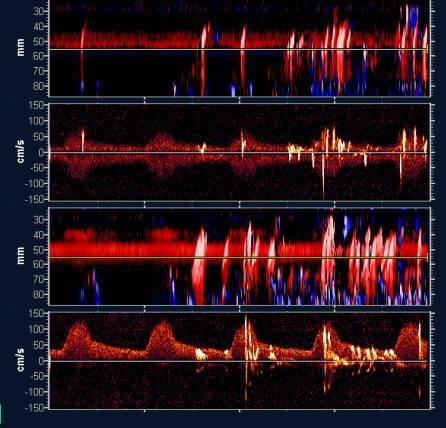
Typical microembolism signals (MES) recorded during cardiac catheterization. Shower of MES during left ventriculography. By convention in our center upper part is for right carotid system and lower part for the left side.

Microembolic signals present a unique signature or "track" in the PMD image, which defines them as representing emboli. These potential embolic tracks in the middle cerebral arteries (MCA, red) and the anterior cerebral arteries (ACA, blue) PMD images have simultaneous spectrogram signatures. However, the spectrogram signatures are restricted to the point in time that the PMD embolic track crosses the gate depth associated with the spectrogram (yellow line). The potential MCA microembolus, which moves toward the probe as time progresses, will have a positively sloped track within the image consistent with its movement direction. The ACA microembolus, which moves away from the probe as time progresses, will have a negatively sloped track within the image. High-power/high-intensity, transient unidirectional signals correspond to the definition of microembolic signature approved by the Consensus Committee of the 9^th ^Cerebrovascular Hemodynamics Symposium [18]. Typical recordings of microembolic signals during cardiac catheterization are shown in Figure 3 and 4.

### Neuropsychological assessment

Patients will undergo neuropsychological assessment within 24 hours before, and 48 hours after, cardiac catheterization. All patients with documented cerebral infarction at DW-MRI will be asked to come back for a new neuropsychological assessment at 1 year follow-up.

The aim of the cognitive testing is to provide clinical evidence of potential cerebral damage after cardiac catheterization and to quantify this potential cognitive decline allowing comparison of two groups of patients explored either by radial or femoral approaches. The tests must be sensitive enough to detect potential post-procedure cognitive decline and also able to explore different brain areas. Most of the neuropsychological tests that will be administered have been reported to be sensitive to change after CABG or PCI procedures [19] and will include: Digit Span Forwards and Backwards, Trail making test A and B, Wechsler Memory Scale visual reproduction test, Rey-Osterrieth's figure test, Grober-Buschckle's test, Rey Auditory Verbal Learning Test, Verbal fluency test, and Confrontation naming test.

### Statistical analysis

Baseline characteristics of the study population will be presented as counts and percents for categorical variables and as mean ± SD for continuous variables. Kappa statistic will be calculated to determine interobserver agreement. The number of lesions in our population of patients recruited will be estimated by the adjusted Wald interval at 95% in accordance with the method recommended by Agresti & Coull [20]. With small sample sizes, as recommended we will use the mid-point of the adjusted Wald interval instead of the observed proportion [21]. The statistical analyses will be performed using SPSS 10.0.7 software (Chicago, IL, USA). The compound score for global cognitive function will be constructed by calculating the average of the z scores for all the above tests. Cognitive decline will be calculated by subtracting the z scores for memory performance, psychomotor speed, and global cognitive function at follow-up from the z scores at base line. All analyses will be adjusted for age, sex, and level of education, as well as the interval between the two sets of neuropsychological tests.

### Sample size calculation

Based on a pilot study [6] we think that radial approach will be associated with a median rate of 5.9% [0.01–12.5;95%CI] of new lesions as detected by cerebral DW-MRI. In patients with aortic stenosis explored by femoral approach we expect a 23% [15–31;95%CI] rate of similar events [3]. With randomization 1:1 an alpha risk of 5% and beta risk of 20% we anticipate a sample size population of 152 patients needed to demonstrate that radial approach could be associated with better outcome concerning the occurrence of silent cerebral infarctions (76 patients in each arm of the study).

### Randomization

After informed consent, eligible patients are randomized 1:1 to a strategy of radial or femoral access. Randomization will be performed at the catheterization laboratory before coronary angiography, by means of randomization list (sealed envelopes) prepared by blocks of 10 patients.

## Discussion

According to previous studies, the rate of stroke after cardiac catheterization ranges from 0.10 % to 0.40 % [22]. However, these studies on the risk of stroke after cardiac catheterization have taken only manifest new neurological deficits as complications. Clinically unapparent damage potentially caused by microscopic air embolism or silent thromboembolism, had not been taken into account. With the advent of DW-MRI, which is very sensitive in detecting acute ischemic lesions within hours after onset [23], it has been shown that asymptomatic embolic events are far more frequent than the apparent neurological complication rate [3-6]. It seems that only the length of the procedure or the procedural fluoroscopy time can be independently associated with the risk of cerebral infarction in these studies. These two parameters are related to the overall influence of the catheter manipulation including additional periods of time while the catheter acts as a embolic source because it may lead to thrombus formation or affect the vessel wall during manipulation or placement in the patient's vascular system. It has also been suggested that silent acute brain injury can be increased using trans-radial access [5] but this has not been demonstrated.

Because all cardiac catheterizations are associated with microembolism as detected by TCD it has been suggested that most of these microembolism might be microbubbles and are therefore likely to be benign. However some recent publications, mentioned above, raise the possibility that some microparticles embolized during heart catheterization could be responsible for acute brain injuries. In fact, the most likely sources of embolic material are catheters and guidewires that dislodge atheromatous material from the aorta [24]. Visible aortic debris may be seen upon withdrawal of catheters during PCI cases [25]. Patients with a large atherosclerotic burden in the aorta as documented by transesophageal echocardiography, such as those with advanced coronary artery disease, have an increased risk of cardiac catheterization-induced stroke [12]. It has been shown that patients with cardiac catheterization-induced stroke often have multiple acute lesions on DW-MRI (often tiny, cortical, and in different vascular territories), distinct from the occasionally symptomatic lesion, and consistent with a shower of embolic material.

Silent cerebral infarction might be a major concern during cardiac catheterization and its potential relationship with cognitive decline needs to be assessed prospectively in a multicenter study. In the SCIPION trial we will use serial DW-MRI to assess the rate of silent cerebral infarction after cardiac catheterization and we will compare the impact of the arterial access site (radial or femoral) on this emerging phenomenon.

## Competing interests

The author(s) declare that they have no competing interests.

## Authors' contributions

All authors have read and approved the final manuscript.
